# Obstruction of the Bowels, of Twelve Days' Standing, Cured by Removing Part of Their Contents by a Syringe

**Published:** 1827-12

**Authors:** James Alexander


CASE II. *Obstruction of the Bowels, of twelve days' standing, cured by removing part of their Contents by a Syringe.* By JAMES ALEXANDER, M.D. (Communicated by Dr. J. JOHNSON.)

I BEG to transmit to you the abstract of a case of intestinal obstruction, successfully treated by, perhaps, an uncommon method, the general knowledge of which may possibly be the cause of some lives being saved, and must certainly diminish the risk many people are occasionally allowed to run, by trusting to, and persevering in, the use of purgatives, however strong, when the stomach and bowels have lost their powers of acting. An insertion of the case into your valuable Journal must be the means of making it known; and, if there are other similar cases, I hope that this, by being added to the stock, and rendering the practice more general, will do some good.

I was called, early on the morning of Friday, the 16th of February, to attend J. G—, esq. commissary-general in his Britannic Majesty's service: he is about fifty years of age, of a spare habit, rather low in stature, and has always been remarkable for temperance and regularity in diet: the only excess, perhaps, of which he has been guilty is that of following the directions of M. Le Roy, and taking large doses of his purgative mixture; but of late he has not taken that medicine,—at least, not within eight months. I think it necessary to premise this, as the risk he ran on the late occasion seemed at first in some degree connected with that torpor and irregular movement in the bowels which the constant use of drastics such as compose Le Roy's mixtures must always produce, and which my patient formerly experienced after their powerful action had ceased.

When I saw him, the account he gave me was this—that for some days previous his bowels had been confined, for which, the day before, he had taken an ounce of manna and ten grains of rhubarb, without any effect; but he nevertheless dined very heartily, and ate with more than usual zest; about half an hour after which he was seized with fainting, great debility, and vomiting, accompanied with a spasmodic pain in the pit of the stomach, extending to the navel. These, in a great measure, subsided upon his being put to bed, and using warm fomentations to the abdomen. In the evening he swallowed an ounce of Castor-oil, which he vomited soon after: with the vomiting he again experienced a return of spasmodic pain in the epigastrium. The fomentations were continued during the night, and at one o'clock the Castor-oil was repeated. At six I first saw him, and found that there had been no movement of the bowels; the pain at intervals continued at the pit of the stomach, but not the least increased on pressure; pulse ninety, and soft. He now showed me a large femoral hernia, the size of an orange, which was tense and elastic, but not at all painful. This he had had during seventeen years, the result of a fall from a horse; it had occasionally increased in size upon the bowels being costive, but was never so large as at this time. By the taxis I reduced it to one-half its usual dimensions (about the size of a hen's egg). He now took another dose of Castor-oil, received an aperient lavement of gruel, oil, and salts; this, and apparently the former dose of medicine, were thrown up; the lavement came away tinged with fæces.

In the course of this day, he vomited frequently a dark-brown matter, highly fetid, apparently the contents of the small intestines. He was also tormented with hiccup to a most painful and alarming degree, with a continual sense of uneasiness and oppression at the precordia.

Four days more he remained in this state, during which I was occupied in combating these various symptoms, and in vain endeavouring to move his bowels, and support his strength. Pills of Scammony, Gamboge, and Colocynth, even his favorite medicine Le Roy, strong enemata, baths, &c. &c. were had recourse to in their turn; and on Monday he had, in divided doses, thirty grains of Calomel and one hundred grains of Jalap, but to no purpose. If the pills were not thrown up, they produced merely a rumbling through about two feet of the canal; after which they seemed to lose their effect, and were no more felt; generally they increased the hiccup and oppression, which went on till by retching he could get rid of them and the other contents of the stomach: then he was somewhat relieved.

On Tuesday morning, by dint of continual exertions, though without inclination, there was a discharge of mucus and a small quantity of feculent matter; but this brought no relief to our patient: on the contrary, all his symptoms increased. He had now great prostration of strength, he was emaciated, his eyes sunk, and his voice much failed.

On Thursday, being unable to procure the Croton-oil, I gave him the oil of Euphorbium (near it in strength, and very active), in a dose of three grains; but he swallowed twenty, in doses of five drops, without feeling them.

I now (Friday) became more alarmed for the fate of my patient, and had a consultation with Dr. Scapi, (a native physician, highly respected, and of great repute in Genoa,) to whom I related the case. He gave little hopes of his recovery; but he recommended a trial of the tobacco glyster, not before used. A pint of the infusion was thrown up three times, but without any effect. In fact, the case seemed quite hopeless, every means had recourse to on similar occasions having so entirely failed.

On Saturday, Mr. G—, fully aware of the approach of his last moments, prepared for them with calm fortitude, taking leave of his family and friends, and had finally arranged his last sad duties: when, in the evening of the twelfth day since having had any passage through his bowels, eight of which he had spent in continual suffering, the idea occurred to me of endeavouring to approach the seat of the evil more nearly; for which purpose I made use of the following means:

I attached a long, hollow, urethra bougie to the pipe of an injection syringe, calculated to contain more than a pint; this I filled with warm water, and having introduced the entire length of the tube by the anus, I threw the contents of the syringe into the bowel with some force. I then attempted to draw back the piston of the syringe, held steadily in the same position, and with some exertion raised it out one-third of its length, for the purpose of withdrawing any fluid within its reach. I had continued this action for about half a minute, when my patient exclaimed that he could not breathe longer, and that something was descending. At this moment I felt a sudden rush of fluid into the body of the syringe, which was very soon filled; and, upon withdrawing the tube, he had immediately a large, feculent, fluid evacuation, attended with instant and complete relief of all his symptoms. He very soon fell asleep, after taking some jelly and wine, which rested comfortably on the stomach: he awakened refreshed, easy, and with an appearance of relief and composure quite new to him for several days. In twelve hours after, he had (as I anticipated) a most violent purging, which seemed to threaten his life, but, by stimulants and nourishment, he rallied again; and, in due time after, his digestive organs resumed their functions, his strength recruited, and I am happy to say he is now restored to perfect health.

I would recommend, in similar cases, an early recourse being had to the tube and syringe, which can be constructed with great ease and expedition as above; but I think the tube might be longer,—say two feet, and of a softer material than the bougie generally is, so as to follow the winding of the intestine. There is nothing unpleasant in its use, more than the common injection-pipe causes, as I have heard from Mr. G—, who, after the end passed some piles, felt nothing; and it was only when I had produced the vacuum in the body of the syringe, and suction in the bowel, that he felt its action. His breathing was then extremely affected, and there was a general bearing down, which continued till he had an evacuation.

*Genoa; March 22d,* 1826.

